# The Effects of Intensity, Exposure Time, and Distance of Polymerization Light on Vickers Microhardness and Temperature Rise of Conventional Resin-Based Composite

**DOI:** 10.3390/polym16223175

**Published:** 2024-11-14

**Authors:** Damir Duratbegović, Nedim Pervan, Selma Jakupović, Sedin Kobašlija

**Affiliations:** 1 Department of Pediatric and Preventive Dentistry, Faculty of Dentistry with Dental Clinical Center, University of Sarajevo, 71000 Sarajevo, Bosnia and Herzegovina; damirduratbegovic@gmail.com (D.D.); skobaslija@sf.unsa.ba (S.K.); 2 Department of Mechanical Design, Faculty of Mechanical Engineering, University of Sarajevo, 71000 Sarajevo, Bosnia and Herzegovina; 3 Department of Restorative Dentistry and Endodontics, Faculty of Dentistry with Dental Clinical Center, University of Sarajevo, 71000 Sarajevo, Bosnia and Herzegovina; jakupovic_selma@yahoo.com

**Keywords:** dental resin-based composites, curing light intensity, distance, exposure time, microhardness, temperature rise

## Abstract

(1) Background: This study investigates the effects of curing light intensity, exposure time, and distance on the Vickers microhardness (VMH), hardness bottom-to-top ratio (HR), and temperature rise (TR) of conventional dental resin-based composite (RBC). (2) Materials and Methods: Specimens of one conventional RBC (Tetric EvoCeram, Ivoclar Vivadent) were cured with 12 different curing protocols (CPs), created with three different light intensities (Quartz Tungsten Halogen 300 mW/cm^2^, LED 650 mW/cm^2^, LED 1100 mW/cm^2^), two exposure times (20 and 40 s), and two distances of curing tip (0 and 8 mm). The VMH of top (VMH-T) and bottom (VMH-B) surfaces was measured. The hardness bottom-to-top ratio (HR) was calculated from VMH-B and VMH-T. The HR below 80% was rated as inadequate polymerization. The TR at the depth of 2 mm within the RBC was measured using a K-type thermocouple. Data were analyzed using Levene’s test and the multivariate analysis of variance (MANOVA). The level of significance was set at *p* < 0.05. (3) Results: Exposure time and distance significantly influenced VMH-B and HR. Increased distance significantly reduced VMH-B, HR, and TR. CPs 300 mW/cm^2^/8 mm/20 s and 650 mW/cm^2^/8 mm/20 s produced inadequate polymerization (HR < 80%). Prolonged exposure time produced higher values of VMH-B and HR. The TR was significantly influenced by light intensity and distance. (4) Conclusions: Suboptimal light intensity (<800 mW/cm^2^) can produce inadequate polymerization at the lower side of the composite layer when used from a distance. Prolonged irradiation can improve the polymerization to a certain extent. Clinicians are advised to monitor the intensity of the LCUs in order to optimize the photopolymerization process. Caution is required when polymerizing with high-intensity curing light in direct contact with the RBC with longer exposure times than recommended.

## 1. Introduction

Resin-based composites (RBCs) are the most used dental restorative material due to their esthetics, durability, cost-effectiveness, versatility, and clinical performance [1]. Composites are a blend of two or more different materials that have different physical and chemical properties, resulting in an overall enhancement of functional properties compared to the individual components [2]. The main components of RBCs are organic matrix resin, inorganic part-fillers, coupling agents, pigments and initiators of polymerization [3,4]. The most commonly used monomers are Bis-GMA, urethane dimethacrylate (UDMA), hydroxyethylmethacrylate (HEMA), triethylene glycoldimethacrylate (TEGDMA), and bisphenol A polyethethylene glycol dimethacrylate (Bis-EMA) [3,4,5]. Inorganic fillers in diverse shapes and sizes are added to enhance the properties of the composites [6]. The organic silane in the coupling agent system improves the bond strength between the filler particles and the resin matrix [5]. When visible blue light (400–500 nm wavelength) is used, the initiator system starts a cross-linking reaction and conversion of carbon double bonds into single bonds to form a stable polymer network [5]. For proper polymerization of the RBC layer and a sufficient degree of conversion of carbon bonds, it is necessary to supply a sufficient amount of radiant exposure [7,8].

Quartz tungsten halogen (QTH) bulbs were used in the earliest light-curing units (LCUs). By the mid-2000s, QTH LCUs were mostly replaced by more efficient light-emitting diode (LED) LCUs due to their production of a higher light intensity, less heat emission, a longer lifespan, and ergonomics [1]. The standard irradiance values of current LED LCUs range from 800 to 2000 mW/cm^2^ with recommended exposure time from 10 to 20 s [1]. In order to further reduce the exposure time from 1 to 5 s, more powerful LCUs (3000–4000 mW/cm^2^) were introduced. The theoretical basis for the reduction in irradiation time is the exposure reciprocity law (ERL), which states that the degree of monomer double-bond conversion in RBCs depends on the delivered radiant exposure energy (J/cm^2^) [9], defined as a product of irradiance (mW/cm^2^) and exposure duration (s) [1]. A radiant exposure of 16 J/cm^2^ is considered sufficient to provide adequate polymerization of a 2-mm-thick increment of RBC [3]. There is still much debate in the field about the effects of applying extremely high irradiance in a short exposure time, and research in this area is still ongoing [1]. The exposure reciprocity is multifactorial and depends on material properties and parameters of curing protocol (CP) [7]. Some researchers have challenged the applicability of ERL [10]. While it may be relevant for certain materials under specific conditions [11,12,13], ERL should not be treated as a universal rule. Instead, it can serve as a guidance for clinicians to adapt the curing duration time to the intensity of the curing light and the clinical conditions in which they perform polymerization (e.g., class II restorations, cusps) [1,8]. According to the ERL, some researchers recommend increasing the duration of irradiance in order to compensate for the increased distance and low light intensity [8,14,15,16]. Considering that a longer exposure time can lead to excessive heat production, some authors recommend that the length of exposure should not last longer than 20 s when using light intensity between 1200 and 1600 mW/cm^2^, and for no longer than 10 s when using light intensity between 2000 and 3000 mW/cm^2^ [17]. Manufacturers of RBCs usually do not give recommendations for the exposure time at increased distances, especially not for suboptimal irradiance (<800 mW/cm^2^).

Inadequate polymerization of RBCs is most often the result of reduced exposure time [14], insufficient light intensity [18], inadequate operator technique [19,20], limited access of the light to the material [21,22], increased distance between the curing tip and the material [15,23], and limitations of the material [24]. Some morphological features in the tooth structure may limit the radiant exposure and energy delivery to the RBCs, as the distance between the cusp height and the cavity floor at class II restorations could reach 8 mm [8]. Although high-intensity LED LCUs have been in clinical use for more than 15 years, studies have shown that there are still LCUs that emit suboptimal light intensity (<800 mW/cm^2^) in daily clinical use [25,26,27,28,29,30,31,32,33]. Moreover, many dentists are not aware of the irradiance levels of their LCUs and often neglect the regular maintenance of these devices [34]. The most common sources of low-intensity curing light in dental practices are quartz tungsten halogen LCUs [25,26,32], outdated and old devices [32,35], weak batteries [36], curing tips covered with debris [33,37], and low-quality LCUs [38]. In order to achieve the best possible properties of the restorative material, clinicians need to follow the manufacturer’s recommendations, be aware of the intensity of the LCU they use, and understand the multifactorial nature of the photopolymerization process.

Multiple in vitro studies have found that prolonged exposure time can produce heat that potentially may adversely affect dental pulp, especially when high irradiance is used [17,39,40,41]. However, the effects of increased distance, low light intensity, and exposure time on the quality of polymerization and thermal changes within RBCs require further study. The microhardness of RBCs can be used to assess the quality of polymerization since the microhardness is related to the degree of conversion as an indicator of the percentage of polymerizable double bonds converted to single bonds [42,43,44,45]. A bottom-to-top hardness ratio (HR) of 80% has been reported to correspond to a bottom-to-top degree of conversion of 90% and is considered a threshold for adequate polymerization [43,45].

The general objective of this study was to evaluate the effects of curing light parameters (suboptimal intensity, exposure time, increased distance) on the quality of polymerization, by measuring the Vickers microhardness (VMH) of the top (VMH-T) and the bottom side (VMH-B) of specimens, bottom-to-top hardness ratio (HR), and temperature rise (TR) during photopolymerization of one conventional RBC.

The null hypotheses of this study were: (i) curing light intensity, distance of curing tip, and exposure time do not affect the VMH-T, VMH-B, and HR of conventional RBC; (ii) prolonged irradiation time cannot improve the VMH-T, VMH-B, and HR of conventional RBC when cured from an increased distance; (iii) prolonged irradiation time cannot improve the VMH-T, VMH-B, and HR of conventional RBC when suboptimal light intensity (<800 mW/cm^2^) is used; (iv) curing light intensity, distance of curing tip, and exposure time do not influence the TR of conventional RBC; (v) prolonged irradiation time does not produce additional TR within the composite, when cured from different distances and with different light intensities ≤ 1100 mW/cm^2^.

## 2. Materials and Methods

### 2.1. Tested Material and Light-Curing Protocols

A conventional nanohybrid resin composite, Tetric EvoCeram (Ivoclar Vivadent AG) was used (Table 1) [46].

Two types of LCUs were used to polymerize the samples: LED LCU (Bluephase Ivoclar Vivadent, Schaan, Lichtenstein) and a QTH LCU (ESPE Elipar II, ESPE Gmbh&Co.KG, Seefeld, Germany). Three values of intensity of curing light were applied: QTH 300 mW/cm^2^ (very low), LED 650 mW/cm^2^ (low), and LED 1100 mW/cm^2^ (standard). Irradiance of the curing light (mW/cm^2^) was controlled after every 5 samples, at the curing tip in direct contact with a digital radiometer, Bluephase meter (Ivoclar Vivadent AG, Schaan, Lichtenstein), and analog radiometer (Spring Light Meter 3 K, Spring Health Products, Norristown, PA, USA). For controlling the distance of the light curing tip from the RBC material, a PVC distance holder of 8 mm in height was used.

Twelve different curing protocols (CPs) applied for the polymerization of composite specimens were combined of 3 different curing light intensities (300, 650, and 1100 mW/cm^2^), 2 distances of curing tip (0 and 8 mm), and 2 exposure times (20 and 40 s) (Table 2). The number of samples for each of the 12 experimental groups was five (*n* = 5). For each of the 12 experimental groups, the Vickers microhardness (VMH), bottom-to-top hardness ratio (HR), and temperature rise (TR) during polymerization were measured.

### 2.2. Vickers Microhardness Specimen Preparation and Measurement Protocol

Sixty disc-shaped specimens (five samples for each curing protocol) were prepared for the Vickers microhardness test, each with a diameter of 4 mm and a thickness of 2 mm. Stainless steel molds were used to shape the samples, which were positioned on a glass slide covered by a Mylar strip. Composite resin was placed into the mold in a single 2 mm increment, then compacted with a Mylar strip and a glass slide, applying static pressure of 0.5 kg for 5 s. After excess material was removed, each sample was photopolymerized from the top side through a Mylar strip, following one of twelve curing protocols. The top surfaces were marked with an indelible marker. Samples were then stored in distilled water at 37 °C in dark containers for 24 h before measurement.

Prior to microhardness testing, both surfaces of each specimen were polished using 2000-grit silicon carbide paper. The Vickers microhardness (VMH) of the top and bottom surfaces was then measured with a Vickers microhardness tester (Clemex CMT.HD, Clemex Technologies Inc., Longueuil, QC, Canada) under a 300 g load and a dwell time of 10 s. Three measurements were taken for each surface, and the average was calculated to yield a single value for each side: VMH-T (top surface) and VMH-B (bottom surface). This value was recorded as the Vicker Hardness Number (VHN).

### 2.3. Bottom-to-Top Hardness Ratio

The bottom-to-top hardness ratio (HR) was calculated as follows:

$$\mathrm{HR}\;(\%)=\frac{VMH\;bottom}{VMH\;top}\times 100$$

According to the accepted standard for bottom-to-top microhardness ratios, an HR above 80% suggests that the polymerization is adequate across the entire composite layer [43,45].

### 2.4. Measurement of Temperature Rise During Photopolymerization

**Figure 1:**
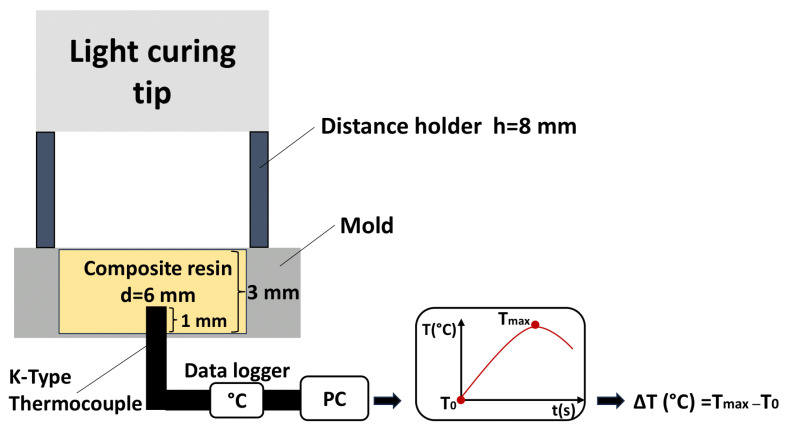
Schematic view of the study design (temperature rise).

A total of 60 disc-shaped composite specimens (5 samples for each curing protocol) with 6 mm in diameter and 3 mm in thickness were prepared in a plastic mold. Every specimen was irradiated with one of 12 curing protocols. Temperature changes were monitored in real time every 1 s during the photopolymerization by means of a K-type thermocouple (Testo 0604 0593, Testo AG, Lenzkirch, Germany), 1.5 mm in diameter and with an accuracy of 0.1 °C. The probe of the thermocouple was positioned centrally at the bottom of composite specimens and immersed 1 mm in the composite layer from a bottom side. The tip of the probe was 2 mm away from the upper surface of the composite material sample. The thermocouple was connected to a data logger (Testo 400, Testo AG, Lenzkirch, Germany), and data were recorded and saved on a computer. The initial temperature at the start of photopolymerization (T_0_) and the highest temperature during photocuring (T_max_) were recorded in °C. The temperature rise (TR) was calculated for each experimental group as the difference between the highest temperature and the initial temperature, TR (°C) = T_max_ − T_0_ (Figure 1). For each curing protocol, five specimens were prepared (*n* = 5).

### 2.5. Statistical Analysis

Statistical analysis was performed using a statistical software (SPSS.13.0, IBM, Armonk, NY, USA). A descriptive analysis and Levene’s test of equality of error variance for independent variables were performed to evaluate the data distribution. Multiple multivariate analysis of variance (MANOVA) tests were used to perform the main results of these analyses. Results were evaluated at a level of *p* < 0.05 significance.

## 3. Results

### 3.1. Vickers Microhardness of the Top Surface (VMH-T)

**Figure 2:**
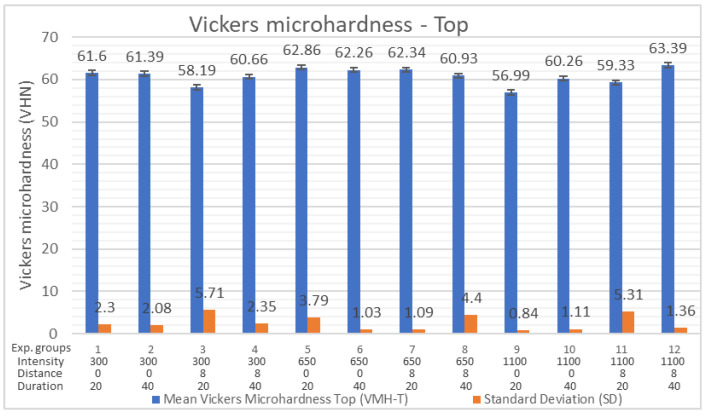
Vickers microhardness of the top surface (VMH-T).

The results of measurement, descriptive statistics, and MANOVA analysis of Vickers microhardness values for the top surface (VMH-T) for all 12 curing protocols are shown in Table 3. The average Vickers microhardness of the top surface of the samples for all 12 experimental groups was 60.85 VHN. The highest average VMH-T (63.39 VHN) was achieved with the curing protocol 1100/8/40. The lowest average VMH-T (56.99 VHN) was achieved with the curing protocol 1100/0/20 (Figure 2). By the multivariate analysis of variance (MANOVA), it was found that there were no significant differences in the VMH-T of samples polymerized with different curing light intensities (*p* = 0.270; *p* = 0.217), different durations of exposure time (*p* = 0.212; *p* = 0.325), and different curing tip distances (*p* = 0.933; *p* = 0.944) (Table 3). VMH-T was not significantly improved by higher light intensity, extended exposure time, or reduced distance.

### 3.2. Vickers Microhardness of the Bottom Surface (VMH-B)

**Figure 3:**
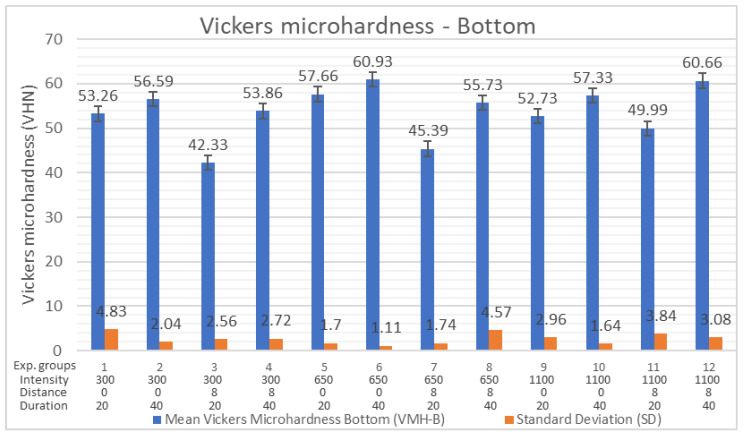
Vickers microhardness of the bottom surface (VMH-B).

The results of measurement and statistical analysis of VMH-B for all 12 curing protocols are shown in Table 4. The total average values VMH-B for all 12 experimental groups was 53.87 VHN and ranged from 42.33 to 60.93 VHN. The highest average VMH-B (60.93 VHN) was achieved with CP 650/0/40. An almost equal value of VMH-B (60.66 VHN) was achieved with CP 1100/8/40. The lowest average VMH-B was produced by CP 300/8/20 (42.33 VHN) and CP 650/8/20 (45.39 VHN) (Figure 3). Multivariate analysis of variance (MANOVA) showed a statistically significant impact of the curing light distance (*p* = 0.024) and exposure time (*p* = 0.004) on VMH-B. Light intensity did not show a significant impact on VMH-B (*p* > 0.05) (Table 4). Decreasing the distance of the light curing tip from 8 mm to 0 mm improved VMH-B regardless of the exposure time (*p* = 0.024). Nevertheless, a descriptive statistic shows that decreasing the distance from 8 mm to 0 mm of the light curing tip emitting light intensity of 1100 mW/cm^2^ does not produce higher values of VMH-B (CP 1100/8 VMH-B = 55.32 VHN; CP 1100/0 VMH-B = 55.03 VHN).

Decreasing the distance from 8 mm to 0 mm with intensities of 300 and 650 mW/cm^2^ produced higher values of VMH-B (CP 300/8 VMH-B = 48.09; CP 300/0 VMH-B = 54.92) (CP 650/8 VMH-B = 50.56; CP 650/0 VMH-B = 59.29) (Table 4, Figure 3). Prolonged exposure time (40 s) significantly increased the values of VMH-B, regardless of tip distance (*p* = 0.004) or light intensity (*p* = 0.051). Although *p* = 0.051 is higher than the standard significance limit of *p* < 0.05, we estimate that this result can be taken as statistically significant because, in other segments of this research, time was also shown to be a statistically significant independent variable (Table 4). The light intensity in this study did not show a significant influence on VMH-B regardless of exposure time (*p* = 0.567) or distance (*p* = 0.625) (Table 4).

### 3.3. Hardness Bottom-to-Top Ratio (HR)

The hardness bottom-to-top ratio (HR) was determined by the values of VMH-B and VMH-T. Since the statistical analysis showed that the tested variables (light intensity, exposure time, and distance) did not show a significant influence on the VMH-T, for the calculation of the HR, the total mean value of the VMH-T (60.85 HV) was used in the denominator. The VMH-B of each sample was brought into relation with the total mean value of VMH-T.

$$\mathrm{HR}\;(\%)=\frac{VMH\;bottom}{VMH\;top(mean\;value)}\times 100$$

**Figure 4:**
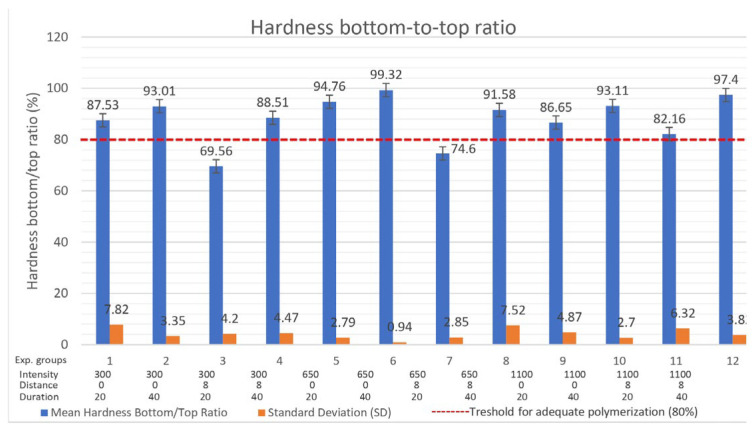
Hardness bottom-to-top ratio (HR).

The highest value, HR = 99.32%, was achieved with curing protocol 650/0/40. The second highest value of HR (97.40%) was achieved with curing protocol 1100/8/40. The minimum hardness ratio of 80%, which was determined as the threshold for adequate polymerization, was not achieved with CP 300/8/20 (HR = 69.56%) and 650/8/20 (HR = 74.60%) (Table 5, Figure 4). Prolonged exposure time (40 s) with intensity of 300 and 650 mW/cm^2^ from a distance of 8 mm produced a significant increase in HR and adequate polymerization (*p* = 0.004). The multivariate analysis of variance (MANOVA) revealed a statistically significant influence (*p* < 0.05) of the distance (*p* = 0.017) and exposure time (*p* = 0.059; *p* = 0.004) on HR (Table 5). Light intensity did not show a statistically significant impact on HR regardless of distance (*p* = 0.651) or exposure time (*p* = 0.614).

When cured from a distance of 8 mm, the use of higher intensity increased the HR, but this difference was not significant (*p* = 0.651) (Table 5). Prolonged irradiation (40 s) produced significantly higher values of HR (*p* = 0.004) regardless of distance (Table 5). From the descriptive statistics, it can be observed that light intensity of 1100 mW/cm^2^ produced almost the same values of HR from both distances (0 and 8 mm) of 89.88% and 89.78%, respectively. A massive decrease in HR values was observed by the increased distance (8 mm) of the curing tip when very low (300/0 HR = 90.27%; 300/8 HR = 79.03%) and low intensity (650/0 HR = 97.04%; 650/8 HR = 83.09%) were used (Table 5). Prolonged exposure time (40 s) produced a significant increase in HR values regardless of light intensity (*p* = 0.059) or curing tip distance (*p* = 0.004). As time is a significant factor in other parts of the study, the value *p* = 0.059, can be taken as significant (*p* ≤ 0.05).

### 3.4. Temperature Rise (TR) During Polymerization

**Figure 5:**
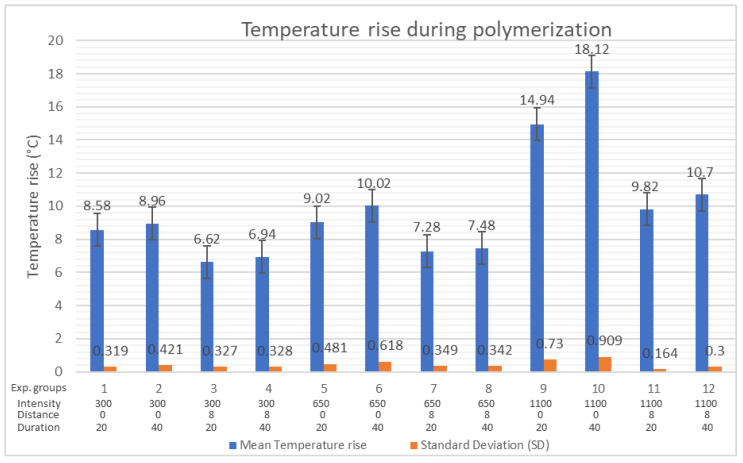
Temperature rise (TR).

The results of statistical analysis of TR for all 12 curing protocols are shown in Table 6. The total average temperature increase (TR) in resin composite specimens during the light polymerization process in all 12 experimental groups was 9.87 °C. The lowest TR was 6.62 °C with CP 300/8/20, and the highest TR was 18.12 °C for CP 1100/0/40 (Table 6, Figure 5). By the multivariate analysis of variance (MANOVA), a strong influence of light intensity and distance was observed. It was found that the intensity of curing light made a significant impact (*p* = 0.000) on temperature rise during polymerization regardless of the distance. The distance of the curing light tip showed a statistically significant impact (*p* = 0.001) on TR, regardless of light intensity (Table 6). Prolonged exposure time did not show a significant impact on TR values, regardless of light intensity (*p* = 0570) or distance (*p* = 0.621). It was observed that longer exposure time produced a higher TR, but these differences were not significant (*p* = 0.062) (Table 6).

Analyzing the means of measured values of VMH-T, VMH-B, HR, and TR in all experimental groups with a light intensity of 1100 mW/cm^2^, and correlating the influence of prolonged irradiation time on VMH and HR with TR, it can be seen that the LED LCU with an intensity of 1100 mW/cm^2^ made the best performance when irradiated 40 s from the distance of 8 mm (VMH-T = 63.39; VMH-B = 60.66; HR = 97.40%; TR = 10.7 °C). In this case, prolonged irradiation time significantly improved the VMH-T, VMH-B, and HR, while this prolongation did not produce a significant amount of additional heat (0.88 °C) within the RBC (Table 7). Lower VMH and HR and higher TR were observed when the light intensity of 1100 mW/cm^2^ was irradiated 20 s from the distance of 0 mm (VMH-T = 56.99; VMH-B = 52.73; HR = 86.65%; TR = 14.92°C), or even 40 s from the distance of 0 mm (VMH-T = 60,62; VMH-B = 57.33; HR = 93.11%; TR = 18.12 °C) (Table 7). When LED light 1100 mW/cm^2^ was used from a distance of 0 mm, prolonged irradiation (40 s) produced a considerably larger amount of additional heat (3.2 °C), in total 18.12 °C.

When the low irradiance (650 mW/cm^2^) was used, higher values of VMH-T, VMH-B, and HR were observed when the curing distance was 0 mm and when the exposure time was extended to 40 s (VMH-T = 62.26; VMH-B = 60.93; HR = 99.32%; TR = 10.02 °C). Increased distance (8 mm) significantly contributed to inadequate polymerization when cured for 20 s (HR = 74.60%). Prolonged irradiation (40 s) from 8 mm significantly improved the quality of polymerization and did not produce a significant amount of additional heat (0.2 °C) (Table 8). Curing protocol 650/0/40 produced almost the same values of (VMH-T, VMH-B, HR, TR) as curing protocol 1100/8/40.

When the very low irradiance (300 mW/cm^2^) was used, higher values of VMH-T, VMH-B, and HR were observed when cured from a distance of 0 mm, with the exposure time of 40 s (VMH-B = 61.39; HR = 93.01%; TR = 8.96 °C). The effects of distance and exposure time on VMH-B and HR were statistically significant and contributed to inadequate polymerization when cured with 300 mW/cm^2^, for 20 s from a distance of 8 mm (HR = 69.56%). Extended exposure time significantly improved the quality of polymerization (HR = 88.51%), and did not significantly increase the temperature rise (0.38 °C at 0 mm; 0.32 °C at 8 mm) (Table 9).

## 4. Discussion

The effective polymerization of RBCs, as indicated by their microhardness, is influenced by various factors, including the parameters of light-curing protocols, and properties of the restorative material [8]. The hardness of the RBCs is defined by the ratio of monomer to polymer conversion; a higher conversion ratio correlates with increased hardness [43]. In the present study, the tested variables of photopolymerization light (light intensity, exposure time, and distance) did not show a significant influence on VMH-T; therefore, for that part of the study, the first hypothesis was accepted. All curing protocols produced roughly the same microhardness of the upper side of the composite layer, independent of light intensity, exposure duration, or distance. Similar studies also stated that the upper surface hardness of resin composites was not dependent on light intensity [47,48,49], distance [50], and exposure time [51]. On the contrary, Tanoue et al. found that the higher light intensity produced higher values of VMH-T [52]. Maximov et al. analyzed the influence of different curing light intensities (600, 1000, 1500 mW/cm^2^) and different curing times (20, 40, 60 s) on the Vickers microhardness of a universal composite and found that light intensity had the most significant influence on the microhardness of the upper side of specimens [53]. However, in the present study, the VMH-B and HR were significantly influenced by the exposure time and distance of the curing tip. Consequently, the first hypothesis was partially rejected for VMH-B and HR in relation to these factors. The Vickers microhardness of the bottom side was significantly higher when the duration of the exposure time was prolonged, and the distance of the light source was reduced. When cured from a distance of 8 mm, the LED light intensity of 1100 mW/cm^2^ achieved better results of VMH-bottom (1100/8 VMH-B = 55.32), than low (650/8 VMH-B = 50.56) and very low irradiance (300/8 VMH-B = 48.09), but these effects were not statistically significant (Table 4). Other studies stated that VMH-B was influenced by light intensity [48], distance [50,54], and duration of exposure [54,55]. Our finding that prolonged exposure from the distance (8 mm) resulted in higher values of the VMH-B, which is similar to the findings of Cekic-Nagas et al., who examined the influence of LED, QTH, and plasma curing units on Vickers microhardness from a distance of 2 and 9 mm [56]. Hasler et al. similarly concluded that the microhardness of the lower side of the composite layer is more influenced by the duration of exposure than by the light intensity [57].

The depth of cure refers to the extent to which the RBC hardens during the light-curing process [58]. A bottom-to-top hardness ratio of 0.80 (80%) or higher is regarded as sufficient for an adequate depth of cure [43,45,58,59]. The depth of cure is influenced by the composition, shade, and translucency of the RBC, as well as the light intensity and the distance from the light source [60]. Our results of HR show that very low QTH light intensity (300 mW/cm^2^) and low LED light intensity (650 mW/cm^2^), when irradiated for 20 s from a distance of 8 mm, produced inadequate polymerization and insufficient microhardness (HR < 80%) of the lower side of a 2-mm-thick composite layer. When cured from a distance of 8 mm, prolonged irradiation time significantly improved the VMH-B and HR of conventional RBC regardless of the light intensity used (Table 7, Table 8 and Table 9); therefore, the second hypothesis of the present study was rejected for VMH-B and HR. When cured with suboptimal (<800 mW/cm^2^) and standard (1100 mW/cm^2^) intensities, prolonged irradiation time significantly improved the VMH-B and HR of conventional RBC regardless of the distance of the curing tip (Table 7, Table 8 and Table 9); therefore, the third hypothesis of the current study was rejected for VMH-B and HR. Curing protocol 650/0/40 produced almost the same values of (VMH-T, VMH-B, HR, TR) as curing protocol 1100/8/40 (Table 7 and Table 8). These findings can be interpreted as a consequence of the exposure reciprocity law (ERL) [9].

Szalewski et al. found that the Vickers microhardness of RBCs is primarily influenced by the duration of exposure, noting that shorter curing times resulted in lower flexural strength and Vickers microhardness values [61]. Other studies also concluded that longer durations of photopolymerization produce higher values of microhardness, regardless of the curing protocol used [62,63]. Par et al. evaluated the effects of prolonged exposure time (30 s) on Vickers microhardness and temperature rise [64]. They found that longer curing durations increase the total energy delivered to composites, which in turn contributes to the higher microhardness and temperature values. This is partly in line with the results of our study, in regard to the microhardness of the bottom side of the composite layer. Peutzfeldt et al. [60] and Cuevas-Suárez et al. [65] found that short exposure times combined with high irradiation powers can result in lower microhardness values compared to LCUs with lower light intensity irradiated with longer curing times, providing that similar total energies are delivered to the material in both scenarios. The duration of curing can significantly affect the Vickers microhardness, with microhardness values increasing when the photopolymerization time is prolonged from 20 to 40 and 60 s, as demonstrated by Hammouda. [66]. Maximov et al. found that curing time is a more important factor than light intensity for the microhardness of the upper and lower sides of a 2-mm universal composite layer [53]. They found that the bottom side has lower microhardness than the upper side and that the curing time has more influence on VMH of the lower side of the universal composite than light intensity [53]. They indicated that differences in factors impacting microhardness on the top and bottom surfaces of the composites are primarily due to the composition of RBC, specifically the monomers in the resin matrix, as well as the type, quantity, and size of the fillers [53]. Jakupović et al. made a similar conclusion, comparing the effects of different high-intensity curing modes on conventional universal and bulk-fill composites [11].

We assume that the variations in microhardness between the upper and lower surfaces of the specimens in our study are attributable to the specific material composition, including a high filler content of 82–83% and filler particle sizes ranging from 40 nm to 3 μm. Additionally, the presence of 3–10% Bis-GMA and 3–10% Bis-EMA, two monomers with a lower degree of conversion, contributes to these differences. The high filler volume fraction and particle sizes, which exceed the wavelength of visible light, result in light scattering and a high attenuation coefficient during curing. These circumstances become especially apparent when using very low (300 mW/cm^2^) and low (650 mW/cm^2^) light intensity from a distance of 8 mm, as in this study. We assume that in two experimental groups (300/8/20 and 650/8/20), the high attenuation of light during passing through the composite layer produced low microhardness values of the bottom side and inadequate polymerization (bottom-to-top hardness ratio < 80%) of the samples. In both cases, by extending the exposure time, the microhardness of the lower side increased (approx. 25%), and adequate polymerization was achieved. This effect of prolonged exposure time was statistically significant and was confirmed by rejecting the second and third null hypotheses of the present research.

In the present study, the temperature rise during the polymerization was significantly influenced by the light intensity and the distance of the curing tip; therefore, the fourth hypothesis of the present study was rejected in terms of light intensity and distance of the curing tip. Rajesh Ebenezar et al. stated that the distance of the curing tip has a significant influence on the TR within resin composites during polymerization [67]. A significant influence of light intensity on the TR was also found in other similar studies [64,67,68,69,70,71,72,73]. In the present study, the greatest TR was recorded for curing protocols 1100/0/40 (18.12 °C) and 1100/0/20 (14.92 °C). Exposure time did not show a significant influence on the TR; therefore the fifth hypothesis of the present study stating that prolonged irradiation time does not produce additional TR within the composite was accepted. Comparing the results of the TR for irradiation times of 20 s and 40 s, it can be seen that the majority (90–95%) of total heat generated in 40 s is generated during the first 20 s. The results of Par et al. showed that the majority of temperature rise occurs within the first 10 s [64]. In the present study, prolonged exposure (40 s) with light intensities of 300 and 650 mW/cm^2^ produced 0.2–1 °C of additional heat in the second half of the curing protocol. When cured from a distance of 8 mm with a light intensity of 1100 mW/cm^2^, prolonged exposure (40 s) produced 0.88 °C of additional heat in the second half of the curing protocol (Table 7, Table 8 and Table 9). However, during polymerization with an intensity of 1100 mW/cm^2^ from a distance of 0 mm, prolonged exposure (40 s) produced 3.18 °C of additional heat in the second half of the curing protocol (TR 20 s = 14.94 °C; TR 40 s = 18.12 °C). Therefore, in order to prevent thermal damage to the pulp, caution is recommended when applying prolonged high-intensity light irradiation in direct contact of the curing tip with the composite material and the tooth (e.g., class V restoration). Other authors also stated that light curing units that emit higher light intensity for extended exposure durations produce more heat compared to those with lower radiant exitance values [39,41]. Other authors similarly recommended that clinicians should limit the exposure time to 20 s when the irradiance from LED units is between 1200 and 1600 mW/cm^2^, and to no longer than 10 s when the LCU irradiance is between 2000 and 3000 mW/cm^2^ [17]. In the present study, the temperature rise means measured at the bottom of composite specimens in all experimental groups ranged between 6.62 and 18.12 °C. In other studies, the temperature rise values vary widely (1.5–23.2 °C) [40,64,74]. This variability in experimental data suggests that quantitative in vitro results cannot be easily interpreted regarding potential risk to the pulp [17,64,75]. In this study, temperature measurements were solely used to evaluate the impact of curing light variables (intensity, duration, and distance) without attempting to correlate the absolute temperature values with the risk of pulpal damage.

A limitation of the present study was that it was conducted in vitro and does not simulate clinical conditions in the oral cavity. The second limitation was that variables of curing light were tested only on one conventional sculptable RBC, since flowable and bulk-fill composites are in daily use in clinical practice. The present study evaluated the influence of curing light with standard (1100 mW/cm^2^) and suboptimal intensities (650 and 300 mW/cm^2^) with exposure times of 20 and 40 s, but in daily clinical use, there are also LCUs with high (2000 mW/cm^2^) and ultra-high (≥3000 mW/cm^2^) light intensities with recommended exposure times from 3 to 10 s. The distance of 8 mm is possible in clinical practice, especially at Class II restorations, but we consider it useful to investigate also smaller distances of curing tip (e.g., 2, 4, 6 mm), since they are more common in clinical practice. Further studies are necessary to assess different aspects of influence of curing light parameters on the polymerization process of RBCs.

## 5. Conclusions

Within the limitations of the present in vitro investigation, the following conclusions can be drawn:

The microhardness of the upper side of the RBC layer is less sensitive to curing light intensity, curing tip distance, and exposure time than the lower side. The microhardness of the lower side of a 2-mm-thick RBC layer is markedly dependent on curing tip distance and exposure time. When the distance between the curing tip and RBC is increased (e.g., class II restorations), curing light with suboptimal light intensity (<800 mW/cm^2^) can produce inadequate polymerization of the lower side of the composite layer, especially if used for an insufficiently long irradiation time. In such conditions, extending the irradiation time can increase the microhardness to a certain extent and improve the quality of polymerization of the lower side of the composite layer. Clinicians are advised to avoid the use of low-irradiance LCUs and to monitor the intensity of the LCUs in order to optimize the photopolymerization process in accordance with the RBC manufacturer recommendations. Temperature rise within RBC during photopolymerization is markedly dependent on the intensity of curing light and the distance of the curing tip. In order to prevent thermal influence on the pulp, caution is required when polymerizing with high-intensity light in direct contact with the RBC with longer exposure times than recommended.

## Figures and Tables

**Table 1:** Resin-based composite used in the study.

Composite Name	Composite Type	Composite Viscosity	Resin Matrix	Filler Type	Filler Content wt%/vol%	Manufacturer
Tetric EvoCeram Shade A2	Universal Conventional	Sculptable	BisGMA *, UDMA **, EthoxylatedBis-EMA ***	Barium glass filler, YbF3, mixed oxide, Prepolymers Size of particles 40 to 3000 nm	82–83% /53–55%	Ivoclar Vivadent AG (Schaan, Liechtenstein)

* BisGMA—bisphenol A glycol dimethacrylate, ** UDMA—urethane dimethacrylate, *** BisEMA—bisphenol A ethoxylate dimethacrylate.

**Table 2:** Curing protocols used in the study.

	Experimental Groups											
	1.	2.	3.	4.	5.	6.	7.	8.	9.	10.	11.	12.
Intensity of curing light (mW/cm^2^)	300				650				1100			
Distance of curing tip (mm)	0	0	8	8	0	0	8	8	0	0	8	8
Exposure time (s)	20	40	20	40	20	40	20	40	20	40	20	40
Curing protocol (intensity/distance/time)	300/ 0/ 20	300/ 0/ 40	300/ 8/ 20	300/ 8/ 40	650/ 0/ 20	650/ 0/ 40	650/ 8/ 20	650/ 8/ 40	1100/ 0/ 20	1100/ 0/ 40	1100/ 8/ 20	1100/ 8/ 40
Number of specimens (*n*)	*n* = 5	*n* = 5	*n* = 5	*n* = 5	*n* = 5	*n* = 5	*n* = 5	*n* = 5	*n* = 5	*n* = 5	*n* = 5	*n* = 5

**Table 3:** Vickers microhardness of the top surface (VMH-T) results and statistical analysis.

Vickers Microhardness-Top (VMH-T)											
Curing Light Variables				Results VMH-Top (VHN) (*n* = 5)							
Light Intensity (mW/cm^2^)	Distance of Curing Tip (mm)	Exposure Time (s)	Exp. Group	1	2	3	4	5	Mean		SD±
300	0	20	1.	63.00	62.00	58.00	61.00	64.00	61.60		2.30
		40	2.	58.66	63.33	59.66	62.66	62.66	61.39		2.08
	8	20	3.	63.66	48.66	58.00	61.00	59.66	58.19		5.71
		40	4.	63.33	59.33	57.33	62.00	61.33	60.66		2.35
650	0	20	5.	65.33	66.33	56.66	63.66	62.33	62.86		3.79
		40	6.	61.33	63.33	63.00	61.00	62.66	62.26		1.03
	8	20	7.	63.66	62.66	62.33	62.66	60.66	62.34		1.09
		40	8.	60.00	59.00	56.66	60.66	68.33	60.93		4.40
1100	0	20	9.	57.00	56.66	56.00	58.33	57.00	56.99		0.84
		40	10.	62.66	60.66	60.00	61.33	60.00	60.26		1.11
	8	20	11.	54.33	54.33	59.66	67.00	61.33	59.33		5.31
		40	12.	65.33	65.66	63.66	63.00	62.66	63.39		1.36
Total mean value (HV)									60.85		
Descriptive statistics VMH-T					Multivariate analysis of variance (MANOVA)						
Light intensity (mW/cm^2^)	Distance of curing tip (mm)	Mean	SD	*n*							
300	0	61.4950	0.14849	2							
	8	59.4250	1.74655	2	Variables		Sum of squares	df	Mean squares	F Value	Signif. *p*
650	0	62.5600	0.2426	2							
	8	61.6350	0.99702	2	Intensity		9.775	2	4.887	1.664	0.270
1100	0	58.6250	2.31224	2	Distance		0.023	1	0.023	0.008	0.933
	8	61.360	2.87085	2	Error		17.835	6	2.972		
Light intensity (mW/cm^2^)	Exposure time (s)	Mean	SD	*n*							Signif. *p*
300	20	59.8950	2.41123	2							
	40	61.0250	0.51619	2	Variables		Sum of squares	df	Mean squares	F Value	
650	20	62.6000	0.36770	2							
	40	61.5950	0.94045	2	Intensity		9.775	2	4.887	1.990	0.217
1100	20	58.1600	1.65463	2	Exposure time		4.788	1	4.788	1.949	0.212
	40	61.8250	2.21324	2	Error		14.736	6	2.456		
Exposure time (s)	Distance of curing tip (mm)	Mean	SD	*n*	Variables		Sum of squares	df	Mean squares	F Value	Signif. *p*
20	0	60.4833	3.09022	3							
	8	59.9533	2.14407	3	Exposure time		4.788	1	4.788	1.100	0.325
40	0	61.3033	1.00281	3	Distance		0.023	1	0.023	0.005	0.944
	8	61.6600	1.50429	3	Error		34.830	8	4.354		

**Table 4:** Vickers microhardness of the bottom surface (VMH-B) results and statistical analysis.

Vickers Microhardness-Bottom (VMH-B)											
Curing light Variables				Results VMH-Bottom (VHN) (*n* = 5)							
Light Intensity (mW/cm^2^)	Distance of Curing Tip (mm)	Exposure Time (s)	Exp. Group	1	2	3	4	5	Mean		SD±
300	0	20	1.	61.00	53.33	49.00	53.66	49.33	53.26		4.83
		40	2.	55.00	59.00	55.00	55.33	58.66	56.59		2.04
	8	20	3.	45.33	38.33	43.33	42.66	42.00	42.33		2.56
		40	4.	54.33	53.00	56.66	55.66	49.66	53.86		2.72
650	0	20	5.	57.66	55.66	56.33	59.66	59.00	57.66		1.70
		40	6.	60.00	59.66	61.66	61.00	62.33	60.93		1.11
	8	20	7.	43.66	45.33	44.00	48.00	46.00	45.39		1.74
		40	8.	53.33	57.00	49.00	59.33	60.00	55.73		4.57
1100	0	20	9.	48.00	53.33	52.33	56.00	54.00	52.73		2.96
		40	10.	54.66	55.33	57.00	58.66	57.66	57.33		1.64
	8	20	11.	50.00	50.66	45.00	55.66	48.66	49.99		3.84
		40	12.	63.33	55.33	62.00	59.00	60.33	60.66		3.08
Total mean value (HV)									53.87		
Descriptive statistics VMH-B					Multivariate analysis of variance (MANOVA)						
Light intensity (mW/cm^2^)	Distance of curing tip (mm)	Mean	SD	*n*							
300	0	54.9250	2.35467	2							
	8	48.0950	8.15294	2	Variables		Sum of squares	df	Mean squares	F Value	Signif. *p*
650	0	59.2950	2.31224	2							
	8	50.5600	7.31148	2	Intensity		33.590	2	16.795	0.508	0.625
1100	0	55.0300	3.25269	2	Distance		77.724	1	77.724	2.351	0.176
	8	55.3250	7.54483	2	Error		198.324	6	33.054		
Light intensity (mW/cm^2^)	Exposure time (s)	Mean	SD	*n*							Signif. *p*
300	20	47.7950	7.72868	2							
	40	55.2250	1.93040	2	Variables		Sum of squares	df	Mean squares	F Value	
650	20	51.5250	8.67620	2							
	40	58.3300	3.67696	2	Intensity		33.590	2	16.795	0.624	0.567
1100	20	51.3600	1.93747	2	Exposure time		159.432	1	159.432	5.921	0.051 *
	40	58.9950	2.35467	2	Error		161.554	6	2.456		
Exposure time (s)	Distance of curing tip (mm)	Mean	SD	*n*	Variables		Sum of squares	df	Mean squares	F Value	Signif. *p*
20	0	54.5500	2.70634	3							
	8	45.9033	3.85571	3	Exposure time		159.432	1	159.432	15.9	0.004 *
40	0	58.2833	2.32175	3	Distance		77.724	1	77.724	7.78	0.024 *
	8	56.7500	3.51288	3	Error		34.830	8	4.354		

* Significant variables.

**Table 5:** Hardness bottom-to-top ratio (HR) results and statistical analysis.

Hardness Bottom-to-Top Ratio (HR)											
Curing Light Variables				Results of Hardness Bottom-to-Top Ratio (%) (*n* = 5)							
Light Intensity (mW/cm^2^)	Distance of Curing Tip (mm)	Exposure Time (s)	Exp. Group	1	2	3	4	5	Mean		SD±
300	0	20	1.	100.0	87.64	80.53	88.18	81.07	87.53		7.82
		40	2.	90.39	96.96	90.39	90.93	96.40	93.01		3.35
	8	20	3.	74.49	62.99	71.21	70.11	69.02	69.56		4.20
		40	4.	89.29	87.10	93.11	91.47	81.61	88.51		4.47
650	0	20	5.	94.76	91.47	92.57	98.04	96.96	94.76		2.79
		40	6.	98.60	98.04	100.0	100.0	100.00	99.32		0.94
	8	20	7.	71.75	74.49	72.31	78.88	75.60	74.60		2.85
		40	8.	87.64	93.67	80.53	97.50	98.60	91.58		7.52
1100	0	20	9.	78.88	87.64	86.00	92.03	88.74	86.65		4.87
		40	10.	89.83	90.93	93.67	96.40	94.76	93.11		2.70
	8	20	11.	82.17	83.25	73.95	91.47	79.97	82.16		6.32
		40	12.	100.0	90.93	100.0	96.96	99.15	97.40		3.82
Total mean value (%)									88.18		
Descriptive statistics VMH-B					Multivariate analysis of variance (MANOVA)						
Light intensity (mW/cm^2^)	Distance of curing tip (mm)	Mean	SD	*n*							
300	0	90.2700	3.87495	2							
	8	79.0350	13.3996	2	Variables		Sum of squares	df	Mean squares	F Value	Signif. *p*
650	0	97.0400	3.22441	2							
	8	83.0900	12.0066	2	Intensity		74.876	2	37.438	0.462	0.651
1100	0	89.8800	4.56791	2	Distance		213.110	1	213.110	2.630	0.156
	8	89.7800	10.7763	2	Error		486.118	6	81.020		
Light intensity (mW/cm^2^)	Exposure time (s)	Mean	SD	*n*							Signif. *p*
300	20	78.5450	12.7067	2							
	40	90.7600	3.18198	2	Variables		Sum of squares	df	Mean squares	F Value	
650	20	84.6800	14.2552	2							
	40	95.4500	5.4730	2	Intensity		74.876	2	37.438	0.530	0.614
1100	20	84.4050	3.17491	2	Exposure time		381.602	1	381.602	5.40	0.059 *
	40	95.2550	3.03349	2	Error		424.034	6	70.672		
Distance of curing tip (mm)	Exposure time (s)	Mean	SD	*n*	Variables		Sum of squares	df	Mean squares	F Value	Signif. *p*
0	20	89.6467	4.45008	3							
	40	95.1467	3.61456	3	Distance		213.110	1	213.110	9.11	0.017 *
8	20	75.4400	6.34186	3	Exposure time		381.602	1	381.602	16.3	0.004 *
	40	92.4967	4.51533	3	Error		186.951	8	23.369		

* Significant variables.

**Table 6:** Temperature rise (TR) results and statistical analysis.

Temperature Rise (TR)										
Curing Light Variables				Results of Temperature Rise (°C) (*n* = 5)						
Light Intensity (mW/cm^2^)	Distance of Curing Tip (mm)	Exposure Time (s)	Exp. Group	1	2	3	4	5	Mean	SD±
300	0	20	1.	8.5	9.0	8.4	8.2	8.8	8.58	0.319
		40	2.	8.8	9.5	8.7	8.5	9.3	8.96	0.421
	8	20	3.	6.5	6.8	6.8	6.9	6.1	6.62	0.327
		40	4.	6.7	7.1	7.3	7.1	6.5	6.94	0.328
650	0	20	5.	9.6	9.4	8.5	8.6	9.0	9.02	0.481
		40	6.	11.0	10.1	9.4	9.6	10.0	10.02	0.618
	8	20	7.	7.3	6.7	7.3	7.6	7.5	7.28	0.349
		40	8.	7.6	6.9	7.5	7.8	7.6	7.48	0.342
1100	0	20	9.	15.1	15.5	14.7	13.8	15.6	14.94	0.730
		40	10.	19.2	18.9	17.3	17.2	18.0	18.12	0.909
	8	20	11.	9.7	10.1	9.8	9.7	9.8	9.82	0.164
		40	12.	11.0	10.4	10.7	11.0	10.4	10.70	0.300
Total mean value (°C)									9.87	
Descriptive statistics VMH-B					Multivariate analysis of variance (MANOVA)					
Light intensity (mW/cm^2^)	Distance of curing tip (mm)	Mean	SD	*n*						
300	0	8.7700	0.26870	2						
	8	6.7800	0.22627	2	Variables	Sum of squares	df	Mean squares	F Value	Signif. *p*
650	0	9.5200	0.70711	2						
	8	7.3800	0.14142	2	Intensity	75.324	2	37.662	37.12	0.000 *
1100	0	16.5300	2.24860	2	Distance	36.053	1	36.053	35.53	0.001 *
	8	10.2600	0.62225	2	Error	6.087	6	1.014		
Light intensity (mW/cm^2^)	Exposure time (s)	Mean	SD	*n*						Signif. *p*
300	20	7.6000	1.38593	2						
	40	7.9500	1.42836	2	Variables	Sum of squares	df	Mean squares	F Value	
650	20	8.1500	1.23037	2						
	40	8.7500	1.79605	2	Intensity	75.324	2	37.662	4.58	0.062
1100	20	12.3800	3.62093	2	Exposure time	2.960	1	2.969	0.360	0.570
	40	14.4100	5.24673	2	Error	49.336	6	8.223		
Exposure time (s)	Distance of curing tip (mm)	Mean	SD	*n*	Variables	Sum of squares	df	Mean squares	F Value	Signif. *p*
20	0	10.8467	3.55175	3						
	8	7.9067	1.68954	3	Exposure time	2.960	1	2.960	0.265	0.621
40	0	12.3667	5.01064	3	Distance	36.053	1	36.053	3.226	0.110
	8	8.3733	2.03296	3	Error	89.418	8	11.177		

* Significant variables.

**Table 7:** Grouped means of all measurements for curing protocols with a light intensity of 1100 mW/cm^2^.

Curing Protocol	VMH-Top	VMH-Bottom	Hardness Bottom-to-Top Ratio (%)	Temperature Rise (°C)
1100/0/20	56.99	52.73	86.65	14.92
1100/0/40	60.26	57.33	93.11	18.12
1100/8/20	59.33	49.99	82.16	9.82
1100/8/40	63.39	60.66	97.40	10.70

**Table 8:** Grouped means of all measurements for curing protocols with a light intensity of 650 mW/cm^2^.

Curing Protocol	VMH-Top	VMH-Bottom	Hardness Bottom-to-Top Ratio (%)	Temperature Rise (°C)
650/0/20	62.86	57.66	94.76	9.02
650/0/40	62.26	60.93	99.32	10.02
650/8/20	62.34	45.39	74.60 *	7.28
650/8/40	60.93	55.73	91.58	7.48

* Hardness bottom-to-top ratio below 80% (inadequate polymerization).

**Table 9:** Grouped means of all measurements for curing protocols with a light intensity of 300 mW/cm^2^.

Curing Protocol	VMH-Top	VMH-Bottom	Hardness Bottom-to-Top Ratio (%)	Temperature Rise (°C)
300/0/20	61.60	53.26	87.53	8.58
300/0/40	61.39	56.59	93.01	8.96
300/8/20	58.19	42.33	69.56 *	6.62
300/8/40	60.66	53.86	88.51	6.94

* Hardness bottom-to-top ratio below 80% (inadequate polymerization).

## Data Availability

The data presented in this study are available on request from the corresponding author.
